# Excision of a Fatty Tumour without Pain

**Published:** 1847-06

**Authors:** 

**Affiliations:** Madras


*Excision of a Fatty Tumour without Pain.* By Dr. JOHNSTONE, of Madras.—Our knowledge of Dr. Johnstone, as well as the inherent correctness of the report, induces us to publish the following case:

Mrs. ——, European, of a well regulated mind, a well formed figure, and a system remarkably free from any kind of nervousness. Has been six years and a half in India. General health good. Before leaving England, she observed a tumour about the size of a field bean over the posterior aspect of the right shoulder. It continued to enlarge gradually but slowly, and at the end of five years had attained the size of a small egg. For the last two years it has increased much more rapidly, and now constitutes a tumour of an adipose nature, lobulated, mobile and kidney-shaped. It measures about six inches in length, four inches in breadth, and two and a half inches in thickness at its thickest part, and stretches from the spinous process of the seventh cervical vertebra, downwards and outwards towards the acromion and outer third of the spine of the scapula, along the upper border of the trapezius muscle. A sensation of weight and slight numbness of the right arm are the chief inconveniences complained of.

Various kinds of treatment having been useless, the patient determined on having it removed. Dr. Johnstone having carefully examined into the evidence brought forward by Dr. Esdaile of Calcutta, in support of the fact of his having performed upwards of one hundred painful surgical operations within the last two years upon natives, under the influence of alleged mesmeric agency; having also read the favourable report of the committee appointed by the honourable the deputy governor of Bengal, to observe and report upon such operations, he advised his patient to try the effect of mesmerism previous to the operation. She consented, and he succeeded on seven following days in putting her into a cataleptic sleep, in which she latterly was unconscious of pinching, pricking with a pin or lancet, and of the manipulation of the tumour. On the eighth occasion, January 9th, 1847, the tumour was removed in the usual way, by means of two elliptical incisions, each seven inches and three lines long, and subsequent dissection of the morbid growth. Three arteries required ligature. The edges of the wound were brought together by four stitches, supported by straps of adhesive plaster. Dr. Smith, Mr. S. S. Young, surgeon, and patient's husband, an apothecary, and a nurse were present. Professor Key had promised to attend, but was unavoidably detained in his class room.

The time of the operation, from the commencement of the first incision to the application of the last roll of bandage, amounted to eighteen minutes, during all of which time, not the slightest trace of suffering or sensibility on the part of the patient could be detected. The pulse continued unchanged at eighty, as S. S. Young satisfied himself, and the respiration perfectly tranquil; no moan or sigh escaped her lips—no alteration in the impression of her features was observed—no instinctive motion or wincing detected; once only she moved her head instinctively to free her mouth and nostrils from a little pool of blood which had collected about them, and was interfering with her breathing. She was easily demesmerized, before which care was taken to conceal as much as possible all traces of the operation. When she awoke the following dialogue ensued. "Well, have you been asleep to-day?" "Yes, I think I have." "Do you think you slept more soundly to-day than yesterday?" "I cannot say." "Did you feel me turn you or do any thing to you to-day?" "No, but I feel something smarting, and my face and my eyes feel stiff." She now put her left hand up to her shoulders as she had often done before, and perceived that the tumour had been removed, of which she confessed perfect unconsciousness. The stiffness of the eyelids and face was caused by dried blood. Pulse eighty—respiration natural.

The tumour weighed 3 lbs. 1 dr. two hours after removal. The wound was dressed with cold dressings, and almost entirely healed up by the first intention. She suffered no pain in the wound, continued perfectly free from fever, and was confined to her room only one day. The pulse continued at eighty for two or three days after the operation, when it rose to ninety, apparently its natural standard. She speedily recovered, and now feels better than she did previous to the commencement of the mesmeric sittings.—*Monthly Journ. of Med. Science.*

